# Maternal Bereavement and Childhood Asthma—Analyses in Two Large Samples of Swedish Children

**DOI:** 10.1371/journal.pone.0027202

**Published:** 2011-11-07

**Authors:** Fang Fang, Caroline Olgart Höglund, Petra Arck, Cecilia Lundholm, Niklas Långström, Paul Lichtenstein, Mats Lekander, Catarina Almqvist

**Affiliations:** 1 Department of Medical Epidemiology and Biostatistics, Karolinska Institutet, Stockholm, Sweden; 2 Respiratory Medicine Unit, Department of Medicine Solna, Karolinska Institutet and Karolinska University Hospital Solna, Stockholm, Sweden; 3 Department of Physiology and Pharmacology, Karolinska Institutet, Stockholm, Sweden; 4 Osher Center for Integrative Medicine, Department of Clinical Neuroscience, Karolinska Institutet, Stockholm, Sweden; 5 Laboratory for Experimental Feto-Maternal Medicine, Department of Obstetrics and Fetal Medicine, University Hospital Hamburg-Eppendorf, Hamburg, Germany; 6 Stress Research Institute, Stockholm University, Stockholm, Sweden; 7 Department of Women's and Children's Health and Astrid Lindgren Children's Hospital, Karolinska University Hospital Solna, Stockholm, Sweden; Leiden University Medical Center, The Netherlands

## Abstract

**Background:**

Prenatal factors such as prenatal psychological stress might influence the development of childhood asthma.

**Methodology and Principal Findings:**

We assessed the association between maternal bereavement shortly before and during pregnancy, as a proxy for prenatal stress, and the risk of childhood asthma in the offspring, based on two samples of children 1–4 (n = 426 334) and 7–12 (n = 493 813) years assembled from the Swedish Medical Birth Register. Exposure was maternal bereavement of a close relative from one year before pregnancy to child birth. Asthma event was defined by a hospital contact for asthma or at least two dispenses of inhaled corticosteroids or montelukast. In the younger sample we calculated hazards ratios (HRs) of a first-ever asthma event using Cox models and in the older sample odds ratio (ORs) of an asthma attack during 12 months using logistic regression. Compared to unexposed boys, exposed boys seemed to have a weakly higher risk of first-ever asthma event at 1–4 years (HR: 1.09; 95% confidence interval [CI]: 0.98, 1.22) as well as an asthma attack during 12 months at 7–12 years (OR: 1.10; 95% CI: 0.96, 1.24). No association was suggested for girls. Boys exposed during the second trimester had a significantly higher risk of asthma event at 1–4 years (HR: 1.55; 95% CI: 1.19, 2.02) and asthma attack at 7–12 years if the bereavement was an older child (OR: 1.58; 95% CI: 1.11, 2.25). The associations tended to be stronger if the bereavement was due to a traumatic death compared to natural death, but the difference was not statistically significant.

**Conclusions/Significance:**

Our results showed some evidence for a positive association between prenatal stress and childhood asthma among boys but not girls.

## Introduction

The prevalence of childhood asthma is high in many countries [1]. The observed marked variation in prevalence among genetically similar populations implies that a substantial proportion of childhood asthma is attributable to environmental factors [2], [3]. Prenatal factors might be important since fetal growth has been suggested to affect an individual's susceptibility to asthma. Prenatal stressors for instance have been linked to negative repercussions on postnatal immune responses of the offspring [4]. Epidemiological studies have indicated that postnatal parental stress is a predictor of asthma and sensitization in infancy [5], [6] and mother's perceived stress is a predictor of childhood wheezing, independent of stress-induced behavior changes including smoking and breast feeding [7]. Maternal stress during pregnancy has also been associated with an increased risk of infant eczema [8]. The influence of prenatal stress on asthma has however rarely been investigated. Bereavement is a unique severely stressful life event and if reasonably strong, the influence of prenatal stress exposure related to a bereavement experience among the expectant mothers on their children could be detected at the population level. For example, based on several national health registers in Denmark, earlier studies have examined associations between maternal bereavement and various health consequences of the children [9], [10], [11], [12], [13], [14].

The aim of this study was to assess the association between maternal bereavement of a close relative from one year before pregnancy to child birth, as a proxy of prenatal stressful experience, and the risk of asthma among the children. The Swedish National Board of Health and Welfare holds several registers covering demographic and health information of the entire Swedish population and it provides a unique opportunity for such analyses. The Personal Identity Number (PIN), a unique identifier for each resident in Sweden, enables unambiguous linkages among these registers as well as other databases held by Statistics Sweden including Death Register and Migration Register.

## Materials and Methods

### Ethics statement

The study was approved by the Regional Ethical Review Board, “Regionala etikprövningsnämnden”, in Stockholm. In accordance with their decision, we did not obtain informed consent from participants involved in the study.

### Asthma definition

Asthma cases were identified from the Swedish Patient Register and Drug Prescription Register. The Patient Register started to collect individual-based information on inpatient care since 1964/1965 and has nationwide coverage of all diagnoses since 1987. Since 2001, this register also covers outpatient visits to specialist care. The Patient Register includes information on one primary diagnosis and up to eight secondary diagnoses for each hospital contact; the *International Classification of Disease (ICD)*-10 has been used for all diagnoses in this register since 1997. The Drug Prescription Register encompasses data on all prescribed medications dispensed in Swedish pharmacies since July 1^st^, 2005. Drugs are coded according to the Anatomical Therapeutic Chemical (ATC) system. All children that had a record in the Patient Register with asthma as the primary or a secondary diagnosis (ICD-10 codes: J45 and J46) or had been prescribed and taken out inhaled corticosteroids or montelukast at least twice were defined as asthma patient. Asthma date was defined as date of the first record in the Patient Register or the first record in the Drug Prescription Register, whichever came first.

### Study participants

The Swedish Medical Birth Register contains data on more than 99% of all births in Sweden since 1973. Starting at the first prenatal visit at the antenatal care clinic, information is prospectively collected on standardized records. We aimed to assess the impact of maternal bereavement on both incident asthma at young age (i.e., first-ever asthma diagnosis as recorded in the Patient Register or a first-ever record of at least two asthma medication dispenses in the Drug Prescription Register at 1–4 years) and any asthma attack among children with potentially established asthma (i.e., a hospital contact for asthma or at least two asthma medication dispenses during a 12-month period at 7–12 years). Accordingly, we studied two samples of children born during July 1^st^, 2004–December 31^st^, 2008 (younger sample; n = 449 363) and January 1^st^, 1997–December 31^st^, 2002 (older sample; n = 514 261) in two respective study periods (younger sample: July 1^st^, 2005–December 31^st^, 2009 and older sample: January 1^st^–December 31^st^, 2009). The study samples and periods were chosen according to the availability of the Drug Prescription Register. Furthermore, since a definitive asthma diagnosis among young children is hard and inhaled corticosteroids or montelukast are widely prescribed for other diagnoses including non-allergic obstructive bronchitis and viral infections of the respiratory tract among young children, we used the term of “asthma event” for asthma and asthma-like event in the younger sample specifically. When talking about both the younger and older samples, the term of “asthma” was used collectively.

We conducted a follow up study for the younger sample where children were followed from their first birthday to the first-ever asthma event, their fourth birthday, death, emigration, or December 31^st^, 2009, whichever came first, through cross linkages to the Patient, Drug Prescription, Death, and Migration Registers. We conducted a cross-sectional analysis in the older sample where a child was termed as an asthma child if he/she had an asthma attack during the year of 2009. A total of 20 742 (4.6%) children from the younger and 16 249 (3.2%) from the older samples that had died, emigrated or had previous asthma event (only applicable to the analysis of the younger sample) before start of the respective study periods were excluded.

### Exposure definition

Exposure was defined as a maternal loss of a close relative (older child, spouse, parent or sibling) from one year before pregnancy to child birth (termed as “maternal bereavement”). Older children of the mothers were identified from the Medical Birth Register and the Swedish Multi-Generation Register (if not born in Sweden). The latter register contains information on all residents in Sweden who were born in 1932 or later and alive in 1961 together with their parents. Spouses were defined as individuals sharing a common biological child with the mothers as recorded in the Multi-Generation Register. Spousal relationship was defined based on an index child and an ex-husband of the child's mother was not counted for that child. Parents and siblings of the mothers were also identified from the Multi-Generation Register. In total, we identified 351 256 older children, 423 440 spouses, 604 929 parents and 472 976 siblings among mothers of the younger sample; as well as 437 793 older children, 493 662 spouses, 680 478 parents and 531 211 siblings among mothers of the older sample.

The relatives were linked to the Causes of Death Register to identify deaths during the defined exposure time window. Time of pregnancy was calculated by date of birth and gestational age. The exposure time window was later divided into within one year before pregnancy and first, second and third trimesters. A total of 7832 children in the younger sample and 10 830 in the older sample were exposed. One loss per mother was counted; when applicable, the hypothesized most severe loss was chosen. The stressfulness of the loss was ranked as: child>spouse>parent or sibling. Children born to the same mothers of the exposed children were excluded from the analyses since they share similar genetic background as the exposed children and might also be influenced by the loss in the family (n = 2287 in the younger and n = 4199 in the older samples), leaving 418 502 and 482 983 unexposed children in the younger and older samples.

### Statistical analysis

For the younger sample we used Cox proportional hazards models to calculate hazard ratios (HRs) of first-ever asthma event and for the older sample logistic regression to calculate odds ratios (ORs) of an asthma attack, comparing exposed to the unexposed children. Since maternal characteristics might be associated with both the maternal risk of bereavement and the child's risk of asthma, we adjusted the estimates for maternal age at delivery (≤19, 20–24, 25–29, 30–34, or ≥35 years), parity status including the index child (1, >1, or missing), educational level (≤9 years, >9 years, or missing), smoking during pregnancy (0, 1–9, ≥10 cigarettes per day, or missing), cohabitation with the child's father (yes, no, or missing), country of birth (Sweden, other Nordic countries, or other countries), as well as BMI at prenatal care registration (<18.5, 18.5–24.9, 25–29.9, ≥30 kg/m^2^, or missing), in addition to child age and gender. Missing was included in the models as a separate group. Since child characteristics at birth might potentially mediate the causal pathway between maternal bereavement and childhood asthma we did not adjust for them in the models. Schoenfeld's partial residuals method suggested little violation of the proportional hazards assumption for any variable but gender of the child and mother's parity status. Accordingly, we stratified all analyses by child gender and mother's parity.

To investigate the potential modifying effect of exposure time window and the magnitude of the stressfulness on the studied association, we broke down the exposed group by the defined smaller time windows, relative type of loss and preparedness of the loss (i.e., natural vs. traumatic death). Traumatic death was defined using the ICD-9 codes 780–799, 807–849, 859–866, 870–929, 950–977, 980–987 and 997–999 (before 1997) and ICD-10 codes R00-R99, V01-X84, Y00-Y36, Y40-Y86, Y870, Y871, Y88 and Y89 (1997 and onward). Statistical analyses were conducted using SAS software version 9.1 (SAS Institute, Cary, NC, USA).

## Results

For both younger and older samples, maternal bereavement was not associated with birth weight, gestational age, gender, or Apgar score at 5 minutes; however, exposed children were more likely delivered via a caesarean section (P<0.0001) (**Table 1**). Mothers of exposed children were older at delivery (P<0.0001), had more likely smoked during pregnancy (P<0.0001) and higher BMI at prenatal care registration (P<0.0001) compared to mothers of other children (**Table 2**).

**Table 1:** Child characteristics in two samples of Swedish children by maternal loss of a close relative due to death from one year before pregnancy to child birth.

	Children born during July 2004–December 2008 (N = 426,334)		Children born during January 1997–December 2002 (N = 493,813)	
	Exposed (n, %)	Unexposed (n, %)	Exposed (n, %)	Unexposed (n, %)
*N*	7832	418,502	10 830	482 983
*Birth weight (grams)*				
≤2999	1017 (13.0)	55 899 (13.4)	1376 (12.7)	61 334 (12.7)
3000–3499	2408 (30.8)	135 521 (32.4)	3184 (29.4)	146 866 (30.4)
3500–3999	2800 (35.8)	147 693 (35.3)	3806 (35.1)	172 167 (35.6)
4000–4499	1256 (16.0)	63 584 (15.2)	1863 (17.2)	79 865 (16.5)
≥4500	337 (4.3)	15 310 (3.7)	544 (5.0)	20 685 (4.3)
Missing	14 (0.2)	495 (0.1)	57 (0.5)	2066 (0.4)
*Gestational age (weeks)*				
≤34	160 (2.0)	7281 (1.7)	215 (2.0)	9169 (1.9)
35–36	250 (3.2)	12 352 (3.0)	351 (3.2)	14 837 (3.1)
37–38	1630 (20.8)	79 706 (19.0)	2136 (19.7)	87 521 (18.1)
39–40	3857 (49.2)	213 983 (51.1)	5393 (49.8)	245 672 (50.9)
41–42	1908 (24.4)	103 798 (24.8)	2658 (24.5)	122 746 (25.4)
≥43	27 (0.3)	1382 (0.3)	77 (0.7)	3038 (0.6)
*Sex*				
Male	3978 (50.8)	212 485 (50.8)	5504 (50.8)	248 583 (51.5)
Female	3854 (49.2)	206 017 (49.2)	5326 (49.2)	234 400 (48.5)
*Apgar score at 5 minutes*				
≤3	8 (0.1)	583 (0.1)	24 (0.2)	1129 (0.2)
4–7	153 (2.0)	7235 (1.7)	194 (1.8)	8415 (1.7)
8–10	7609 (97.2)	407 357 (97.3)	10 476 (96.7)	467 933 (96.9)
Missing	62 (0.8)	3327 (0.8)	136 (1.3)	5506 (1.1)
*Mode of delivery*				
Vaginal delivery (VD)	6258 (79.9)	349 247 (83.4)	9007 (83.2)	415 516 (86.0)
Caesarean section (CS)	1574 (20.1)	69 255 (16.6)	1823 (16.8)	67 467 (14.0)

**Table 2:** Mother characteristics at delivery in two samples of Swedish children by maternal loss of a close relative due to death from one year before pregnancy to child birth.

	Children born during July 2004–December 2008 (N = 426,334)		Children born during January 1997–December 2002 (N = 493,813)	
	Exposed (n, %)	Unexposed (n, %)	Exposed (n, %)	Unexposed (n, %)
*N*	7832	418,502	10 830	482 983
*Mother's age (years)*				
≤19	79 (1.0)	7173 (1.7)	121 (1.1)	9208 (1.9)
20–24	609 (7.8)	52 039 (12.4)	881 (8.1)	70 046 (14.5)
25–29	1728 (22.1)	121 284 (29.0)	2729 (25.2)	166 835 (34.5)
30–34	2850 (36.4)	150 943 (36.1)	3962 (36.6)	159 446 (33.0)
≥35	2565 (32.8)	87 025 (20.8)	3137 (29.0)	77 445 (16.0)
Missing	1 (0.0)	38 (0.0)	-	3 (0.0)
*Parity*				
1	2857 (36.5)	191 155 (45.7)	3565 (32.9)	209 768 (43.4)
>1	4975 (63.5)	227 346 (54.3)	7265 (67.1)	273 196 (56.6)
Missing	-	1 (0.0)	-	19 (0.0)
*Education (years)*				
≤9	798 (10.2)	43 966 (10.5)	1511 (14.0)	62 697 (13.0)
>9	6984 (89.2)	364 740 (87.2)	9237 (85.3)	409 072 (84.7)
Missing	50 (0.6)	9796 (2.3)	82 (0.8)	11 214 (2.3)
*Smoking during pregnancy (cigarettes per day)*				
0	6535 (83.4)	361 864 (86.5)	8399 (77.6)	396 041 (82.0)
1–9	563 (7.2)	21 958 (5.2)	1054 (9.7)	38 326 (7.9)
≥10	198 (2.5)	6865 (1.6)	630 (5.8)	17 498 (3.6)
Missing	536 (6.8)	27 815 (6.6)	747 (6.9)	31 118 (6.4)
*Cohabits with child's father*				
Yes	6921 (88.4)	372 495 (89.0)	9610 (88.7)	432 474 (89.5)
No	422 (5.4)	21 712 (5.2)	568 (5.2)	23 025 (4.8)
Missing	489 (6.2)	24 295 (5.8)	652 (6.0)	27 484 (5.7)
*Country of birth*				
Sweden	7242 (92.5)	331 814 (79.3)	10 223 (94.4)	401 830 (83.2)
Other Nordic countries	72 (0.9)	7069 (1.7)	144 (1.3)	10 026 (2.1)
Other	518 (6.6)	79 619 (19.0)	463 (4.3)	71 127 (14.7)
*BMI (kg/m^2^)*				
<18.5	144 (1.8)	8907 (2.1)	175 (1.6)	10 476 (2.2)
18.5–24.9	3961 (50.6)	229 938 (54.9)	5498 (50.8)	260 579 (54.0)
25–29.9	1884 (24.1)	92 476 (22.1)	2394 (22.1)	101 116 (20.9)
≥30	994 (12.7)	42 263 (10.1)	1147 (10.6)	40 234 (8.3)
Missing	849 (10.8)	44 918 (10.7)	1616 (14.9)	70 578 (14.6)

A total of 537 asthma cases were observed among the exposed children in the younger and 397 in the older samples (**Table 3**). In the younger sample, the crude incidence rate of asthma event was slightly higher among the exposed children if the bereavement happened during the year before pregnancy or the second trimester, compared to unexposed children. The mean age of asthma event did not vary by exposure status in the younger sample. In the older sample, the percentage of children that had an asthma attack during 2009 was slightly higher among exposed children compared to the unexposed children.

**Table 3:** Asthma outcomes in two samples of Swedish children by maternal loss of a close relative due to death from one year before pregnancy to child birth.

	Children born during July 2004–December 2008 (N = 426 334)				
	Exposed (N = 7832)				Unexposed
	1 yr before pregnancy	1^st^ trimester	2^nd^ trimester	3^rd^ trimester	
N	4594	934	954	1350	418 502
Male gender, n, %	2355 (51.3)	468 (50.1)	463 (48.5)	692 (51.2)	212 485 (50.8)
No. with asthma event*	325	60	74	78	26 268
Person-years	8696	1780	1866	2643	784 536
Incidence rate†	37.37	33.71	39.66	29.51	33.48
Asthma age(yrs), mean (SD)	1.83 (0.67)	1.91 (0.64)	1.85 (0.60)	1.84 (0.69)	1.85 (0.68)

	Children born during January 1997–December 2002 (N = 493 813)				
	Exposed children (N = 10 830)				Unexposed
	1 yr before pregnancy	1^st^ trimester	2^nd^ trimester	3^rd^ trimester	
N	6393	1278	1343	1816	482 983
Male gender, n, %	3219 (50.4)	658 (51.5)	694 (51.7)	933 (51.4)	248 583 (51.5)
No. with asthma attack*	242	46	41	68	16 665
Prevalence, %	3.78	3.60	3.05	3.74	3.45

*defined as a hospital contact for asthma or two dispensed prescriptions of inhaled corticosteroids or montelukast;

† per 1000 person-years.

Overall, exposed children did not have a higher risk of asthma compared to unexposed children (younger sample: HR: 1.06; 95% CI: 0.97, 1.15 and older sample: OR: 1.06; 95% CI: 0.95, 1.17). A positive association was clearer among boys but totally null among girls (**Table 4**). We therefore presented results among boys and girls separately. A statistically significantly higher risk of first-ever asthma event was observed among boys exposed to maternal bereavement during second trimester in the younger sample, but not for an asthma attack in the older sample. A higher risk of asthma was also suggested for boys exposed to a maternal bereavement of an older child, especially for the older sample. Compared to bereavement of natural causes, a bereavement of traumatic causes appeared to be more strongly associated with a higher risk of asthma, but the difference was not statistically significant. A maternal loss of spouse, for both boys and girls, appeared to be associated with a higher risk of an asthma attack at 7–12 years, but the estimates were not statistically significant.

**Table 4:** Relative risks of asthma among children exposed to maternal loss of a close relative due to death from one year before pregnancy to child birth, compared to unexposed children.

	Children born during July 2004–December 2008 (N = 426 334)		Children born during January 1997–December 2002 (N = 493 813)	
	No. of cases	Hazard ratio (95% CI)*	No. of cases	Odds ratio (95% CI)*
**Boys**				
Unexposed	15 601	1.00	10 575	1.00
Exposed	332	1.09 (0.98–1.22)	258	1.10 (0.96–1.24)
By time window				
≤1 year before pregnancy	203	1.13 (0.99–1.30)	155	1.14 (0.96–1.34)
1^st^ trimester	34	0.92 (0.66–1.30)	28	0.98 (0.67–1.43)
2^nd^ trimester	55	1.55 (1.19–2.02)	27	0.89 (0.61–1.31)
3^rd^ trimester	40	0.75 (0.55–1.02)	48	1.20 (0.90–1.60)
By relative type				
Older child	38	1.26 (0.92–1.73)	33	1.58 (1.11–2.25)
Spouse	2	0.47 (0.12–1.87)	4	1.90 (0.68–5.29)
Parent	269	1.07 (0.95–1.21)	207	1.05 (0.91–1.21)
Sibling	23	1.21 (0.80–1.82)	14	0.94 (0.55–1.60)
By cause of death				
Traumatic	57	1.24 (0.95–1.60)	36	1.36 (0.97–1.90)
Natural	275	1.06 (0.94–1.20)	222	1.06 (0.93–1.22)
**Girls**				
Unexposed	10 667	1.00	6090	1.00
Exposed	205	1.00 (0.87–1.15)	139	0.99 (0.84–1.18)
By time window				
≤1 year before pregnancy	122	1.03 (0.86–1.24)	87	1.04 (0.84–1.30)
1^st^ trimester	26	1.07 (0.73–1.57)	18	1.10 (0.69–1.76)
2^nd^ trimester	19	0.70 (0.44–1.09)	14	0.82 (0.48–1.40)
3^rd^ trimester	38	1.06 (0.77–1.46)	20	0.85 (0.55–1.33)
By relative type				
Older child	19	0.98 (0.62–1.54)	14	0.99 (0.58–1.69)
Spouse	3	1.25 (0.40–3.87)	4	2.66 (0.96–7.37)
Parent	169	1.00 (0.86–1.16)	111	0.95 (0.79–1.15)
Sibling	14	0.97 (0.58–1.64)	10	1.28 (0.68–2.40)
By cause of death				
Traumatic	29	0.94 (0.65–1.35)	16	1.14 (0.69–1.87)
Natural	176	1.01 (0.87–1.17)	123	0.98 (0.81–1.17)

*CI = confidence interval. Models were adjusted for age and gender of child, maternal age at delivery, country of birth, parity status, education level, smoking during pregnancy, cohabitant status with child's father, and BMI at perinatal care registration.

## Discussion

Based on two large samples of Swedish children, we found some evidence of a weak association between maternal bereavement experienced from one year before pregnancy to child birth and childhood asthma among boys while not girls. The associations also tended to be stronger for loss of a child or loss due to a traumatic cause of death.

Given that asthma has a strong immunological component; it is tempting to speculate that the fetal immune system altered upon prenatal stress challenges may contribute to the etiopathogenesis of asthma. It is well established that the immune system in mammals begins to develop prenatally [15], [16], [17] with thymus organogenesis and T cell development in the thymus as a milestone [18]. It is hypothesized that prenatal stress, through HPA-axis activation and fetal programming [19], modulates the developing immune system with enhanced polarization towards Th2 phenotype. For instance, stress-induced alterations in maternal cortisol may influence fetal immunomodulation and Th2 cell predominance through a direct influence on cytokine production [5]. Further, the development of regulatory T cells may be impaired [20], [21] and stress may impact the maturation process of dendritic cells, further predisposing to a Th2 phenotype [22]. Although there is few data on humans, animal models showed that prenatal psychological stress increases the risk of chronic immune diseases including various allergies and vulnerability toward airway hyperresponsiveness [23]. These effects are probably due to stress-induced altered activity of HPA-axis which has immunoregulatory effects on the expression of IgE during pregnancy [19], [24]. A recent study also showed that newborns to mothers that had experienced a stressful period during pregnancy exhibited different innate and adaptive immune response [25]. Finally, altered neuroimmune responsiveness may also influence the expression of asthma by enhancing an individual's susceptibility to other environmental factors which may contribute to asthma risk independently [26].

The stronger impact of loss of a child or of a traumatic cause, compared to loss of other relatives or loss due to more natural causes, on childhood asthma seems to be in agreement with previous findings on maternal bereavement and other childhood diseases [9], [10], [14], [27]. The different results between boys and girls as shown in our data are intriguing. Although the possibility of chance finding could not be ruled out, gender specific association between maternal bereavement and childhood diseases have also been described earlier. For example, a positive association between maternal bereavement and attention-deficit/hyperactivity disorder was mainly among boys but not girls [10], whereas for type 1 diabetes mainly among girls but not boys [14]. A gender specific association may also be plausible for asthma given that gender differences in asthma are well established - boys have a higher prevalence of wheezing and asthma than girls before puberty but the pattern shifts toward girls around and after puberty [28]. Boys have also been described to have different asthma-related traits such as bronchial hyperresponsiveness, allergic sensitization, serum IgE levels and developmental cytokine response profiles from girls [29], [30], [31]. There are also differential growth of lung/airway size and immunological profiles between boys and girls [32], [33]. With respect to the development of asthma, these gender differences could all potentially make boys more sensitive to the impact of stress hormones as compared to girls.

Strengths of our study include the nationwide population-based design in a unified health care environment, prospectively and independently collected information on exposure and potential confounders that preclude recall bias, and the ascertainment of asthma by applying predetermined asthma criteria to the Patient Register and Drug Prescription Register. Sweden offers free inpatient and outpatient medical care to all residents. The Patient Register covers inpatient and outpatient visits but not visits to general practitioners and therefore we included asthma medication use from the Drug Prescription Register as an additional asthma ascertainment strategy in the present analyses where all dispensed asthma medications (except for those used during hospitalization) are registered. By using these asthma ascertainment criteria, we believe that we were able to identify the vast majority of asthma cases that had come into notice of the health care system. Furthermore, we were also able to study both incident cases of asthma symptoms among younger children (at 1–4 years) and any asthma attack among children with likely established diagnosis (at 7–12 years). Defining asthma is difficult among young children and use of inhaled corticosteroids and montelukast is not greatly specific for asthma. However, we believe that we have captured the real asthma cases (e.g., through the Patient Register) or the relatively severer cases of other conditions (i.e., with at least two dispenses of these specific medications) that will quite likely develop as asthma later on given that frequent wheezing such as obstructive bronchitis or (viral) infections of the respiratory tract during the first few years of life is a strong risk factor for subsequent asthma symptoms [34]. In our multivariable analysis, maternal age at delivery, smoking during pregnancy and BMI at the prenatal care registration were all shown to be associated with a higher risk of asthma (data not shown), which further supported the validity of our asthma definition.

One inherent limitation of the present study, as of other register-based studies, is potential residual confounding due to factors not recorded in the registers. For example, although bereavement, especially loss of a child, is the most severe stressful life event that one may encounter, pregnant women might experience a variety of other milder stressors which could theoretically lead to an under-estimated association in our study. We also lacked information on other maternal characteristics including diet, physical activity and health seeking behaviors that might be related to both the mother's perception of the bereavement and the child's probability of being identified as an asthma patient by the health care system. Further, although we adjusted for maternal characteristics in the analyses, changes in social and economic status of the family (or mothers) after child birth as a result of the bereavement which might result in different asthma risk of the children were not captured. For example, loss of a child due to death has been associated with a higher risk of hospitalization for psychiatric disorders among the parents especially mothers [35]. It is possible that these health consequences may result in less successful career of the parents and less affluent living environment among the children. Our findings that maternal loss of a child and spouse appeared to be the strongest risk predictors for child asthma at 7–12 years, compared to maternal loss of a parent or sibling, agreed with this line of reasoning. On the other hand, this possibility was not backed up by the stronger impact of a traumatic bereavement compared to bereavement of a natural cause. If financial setback were a mediate for the studied association, bereavement of natural causes such as a chronic disease should be more influential given the care giving burden of the family in addition to the psychological stress. Finally, although with the large material, our study was still underpowered to examine the potential modifying effect of timing and relative type of loss on the studied association.

In brief conclusion, our study provided some evidence for a potential association between prenatal stress and a higher risk of childhood asthma, mainly among boys.
